# Improving Imitation Skills in Children with Autism Spectrum Disorder Using the NAO Robot and a Human Action Recognition

**DOI:** 10.3390/diagnostics15010060

**Published:** 2024-12-29

**Authors:** Abeer Alnafjan, Maha Alghamdi, Noura Alhakbani, Yousef Al-Ohali

**Affiliations:** 1 Computer Science Department, College of Computer and Information Sciences, Imam Mohammad Ibn Saud Islamic University (IMSIU), Riyadh 11432, Saudi Arabia; 2 Information Technology Department, College of Computer and Information Sciences, King Saud University, Riyadh 11543, Saudi Arabia; 3 Computer Science Department, College of Computer and Information Sciences, King Saud University, Riyadh 11543, Saudi Arabia

**Keywords:** robot, autism, therapy, human action recognition, imitation, convolutional neural network

## Abstract

**Background/Objectives:** Autism spectrum disorder (ASD) is a group of developmental disorders characterized by poor social skills, low motivation in activities, and a lack of interaction with others. Traditional intervention approaches typically require support under the direct supervision of well-trained professionals. However, teaching and training programs for children with ASD can also be enhanced by assistive technologies, artificial intelligence, and robotics. **Methods:** In this study, we examined whether robotics can improve the imitation skills of children with autism and support therapists during therapeutic sessions. We designed scenarios for training hand clapping imitation skills using the NAO robot and analyzed the interaction between children with autism and the robot. **Results:** We developed a deep learning approach based on the human action recognition algorithm for analyzing clapping imitation. **Conclusions:** Our findings suggest that integrating robotics into therapeutic practices can effectively enhance the imitation skills of children with ASD, offering valuable support to therapists.

## 1. Introduction

According to the Diagnostic and Statistical Manual of Mental Disorders, autism spectrum disorder (ASD) is a serious disability causing functional impairment in social interactions and communication across various contexts. ASD is often characterized by restricted stereotypical or repetitive motor activities [1].

Children with ASD cannot easily recognize body language, make eye contact, explain personal feelings, or understand other people’s emotions. They also present obvious difficulties in imitation skills, having a lower frequency of imitation skills than that of typically developing children. Imitation includes body movement, whispering, and facial expressions through which children communicate with or oppose their parents [2]. Improving imitation skills during the early stages of development is essential for improving communication [3]. Faced with these limitations, health and educational professionals frequently struggle to motivate and engage children with ASD in treatment and learning activities. Participation in diverse social, playing, educational, and therapeutic activities is essential for acquiring the knowledge necessary for cognitive and social development [2].

Recently, robots have assisted various human tasks and the rehabilitation of individuals with ASD [4]. Involving social or interactive robots in clinical practice appears to be promising for improving the social skills of children with ASD. Humanoid robot-assisted teaching and intervention programs for children with ASD are rapidly evolving [5]. According to the Gartner’s 2023 Hype Cycle report on artificial intelligence (AI) trends [6], smart robots have reached the peak of the inflated expectation stage and their mainstream adoption is expected in 5–10 years.

Traditional intervention approaches usually require intensive support under the direct supervision of well-trained professionals. Nevertheless, many individuals with autism cannot continuously access professional care and amenities owing to high intervention costs and a lack of available qualified therapists [7].

Several interventions have attempted to improve the cognitive ability and daily living skills, increase the interaction and engagement in the community, and reduce the symptoms of children with ASD. For example, therapy sessions have adopted assistive technologies to support and improve current therapies for the increasing number of children with ASD [8].

To analyze the behavior and engagement levels of children with autism during therapy, Belpaeme et al. [9] input sensory features such as facial expression, body movements, and voice recordings to a machine learning model implemented in a robot. These input features were then merged with engagement markers for model training.

Wan et al. [10] proposed a novel framework for human–computer/robot interaction and introduced a preliminary intervention study for improving emotion recognition in Chinese children with ASD. Deep learning algorithms were deployed for facial expression recognition and attention analysis at the back end of the system. The analysis of the emotion-learning progress of these children with autism could be accessed by parents and therapists through a cloud-based evaluation system.

Studies have shown that AI-assisted tools exert a positive impact and are acceptable to teachers, parents, and therapists; moreover, they can be feasibly implemented in teaching or therapeutic practices. Therefore, machine learning can potentially improve the social interaction skills and supportive education of children with mental disorders [11].

In this study, we implemented robotics to improve the imitation skills of children with autism in Saudi Arabia and assist therapists during therapy sessions. We designed scenarios for training imitation skills in children with autism using the NAO robot, and then analyzed the interaction between the children with autism and the robot using the human action recognition (HAR) algorithm. Finally, we evaluated the learning progress and compared the effectiveness of robot-assisted therapy and traditional therapy in developing imitation skills in children with autism. This exploratory study is primarily based on AI and robotics. The aim is to extend motion detection using robotics to the context of autism therapy.

The remainder of this paper is structured as follows. Section 2 presents the necessary interdisciplinary background of this research, overviewing the most relevant fields of study discussed herein. Section 3 illustrates the system framework and technology of the present study. Section 4 details the implementation of the project. Section 5 presents the experimental design and all related information. Section 6 concludes the paper and makes recommendations for future research.

## 2. Background

This section provides the necessary theoretical background for our study. After discussing ASD, we briefly overview assistive technologies, particularly socially assistive robotics (SAR), Robot-Assisted Imitation Skills, and then present HAR.

### 2.1. Autism Spectrum Disorder (ASD)

ASD refers to a group of early-appearing social communication impairment and repetitive sensorimotor behaviors. ASD has a strong genetic component and can be triggered by other causes [12]. The core underlying feature of ASD is a severe deficit in social reciprocity skills. Regardless of their cognitive or language abilities, people with ASD are majorly impaired by socialization deficits. As children approach adolescence, their deficits and distress may increase as the social environment becomes more complex and the child becomes more aware of their social disability [13].

Early indications of ASD can be difficult to recognize for several reasons. First, early ASD symptoms widely vary in type and severity. Furthermore, early signs of ASD can occur in children without ASD, and some common early signs can be absent in children with ASD. Infants with ASD typically reach their physical developmental milestones (rolling over, sitting up, crawling, and walking) at a normal rate, making it difficult for parents and healthcare providers to detect ASD’s subtle impact on the development of social and communication skills [14].

Individuals with ASD may behave, communicate, interact, and learn differently compared to most other people, although their appearance is often indistinguishable from that of others. People with ASD also display various abilities. For example, some people with ASD have advanced conversation skills while others are nonverbal [15]. Some people with ASD require much assistance in their daily lives, while others can work and live with little or no assistance. The Diagnostic and Statistical Manual of Mental Disorders (DSM-5) [1] recognizes three levels of autism based on two areas of functioning (social communication and restricted, repetitive behaviors): Level 3 requires very substantial support, Level 2 requires substantial support, and Level 1 requires standard support.

Current ASD treatments aim to reduce the symptoms that interfere with daily functioning and quality of life. Because the effects of ASD differ among individuals, people with ASD have unique strengths and challenges, along with different treatment needs. Therefore, treatment plans are tailored to the individual and usually involve several specialists [15].

### 2.2. Socially Assistive Robotics (SAR)

The perception of assistive technology largely determines the acceptability of this technology. SAR attempts to improve the social interaction between users and assistance robots in social interaction settings [16]. Social robots can perform diverse interactions that meet the clinical needs of users with no or minimal involvement of a professional. Consequently, SAR can monitor the user’s treatment progress while simultaneously providing education and feedback to the user [17].

Social robots are broadly classified into socially evocative, social interface, socially receptive, and sociable robots [18,19]. Socially evocative robots entertain users through interactive sessions with toys and other playful mechanisms. Social interface robots (such as airport guides) simulate human-like conversation along with body language and facial expressions. Socially receptive robots interact with and learn from humans to acquire social intelligence skills. Sociable robots actively engage in social situations to fulfill their own social goals [18,19].

The utilization of socially evocative robots for the diagnosis and treatment of autism has gained traction over the past decade. Studies exploring the potential use of SAR in autism treatment have reported that socially evocative robots enhance the engagement, attention, and social skills of users. Furthermore, some children with autism might benefit from robotic interactions because robot behavior is more predictable and consistent than human behavior [20].

### 2.3. Robot-Assisted Imitation Skills

Numerous studies have explored the use of robots to support the growth and development of children with ASD by integrating therapy and education. These interventions target essential learning skills, including attention, joint attention, social referencing, imitation, and both receptive and expressive language, particularly crucial in the early stages of treatment [4].

Imitation plays a vital role in childhood learning, influencing play, social interaction, and the acquisition of socio-emotional understanding [2]. The development of imitation skills in children with ASD significantly impacts various social and cognitive domains. Enhanced imitation allows these children to better observe and replicate social behaviors, improving their understanding of social norms and interactions, which in turn fosters better communication through mimicking speech patterns and emotional expressions. This skill also contributes to the development of empathy and theory of mind, enabling them to gain insights into others’ perspectives and feelings, thereby enhancing social engagement. Additionally, imitation facilitates cognitive development by aiding problem-solving and creativity, as children learn new strategies through observation. It strengthens social bonds by promoting shared activities and connections, and it supports behavioral regulation by helping them adapt their behaviors to social cues [3].

For instance, Conti et al. [11] provide empirical evidence for robot-assisted imitation training, demonstrating that a robotic assistant can be effectively integrated into the standard treatment protocols for children with varying degrees of intellectual disabilities. Post-training, children showed significant improvements in gross motor imitation skills. Telisheva et al. conducted quantitative analyses of ASD children’s interactions with robots during play-based activities. These activities encompassed tasks related to imitation, turn-taking, emotions, and other social skills. Their findings suggest that robots can engage socially with children and positively influence their social behaviors over time.

### 2.4. Human Action Recognition (HAR)

Over the last decade, HAR has been actively researched in the computer vision field [21]. Herath et al. [22] defined action as “the most elementary human-surrounding interaction with a meaning”. The HAR process labels the actions of a human agent within a given sequence of images to classify the agent’s goals in a series of image frames. Action recognition typically aims to discover a class of short, segmented atomic actions [23].

In general, HAR is a hierarchical process in which the lower levels perform human detection and segmentation by identifying the regions of interest corresponding to static or moving humans in a video. In the upper levels, visual information of the action is extracted and represented by features for subsequent action recognition. Such feature-based action recognition can be viewed as a classification problem [24].

HAR categorization has remained a difficult task in computer vision. HAR methods have been categorized into two main categories, namely, unimodal and multimodal, depending on the nature of their sensor data [25]. Unimodal HAR methods identify human activities from the data of a single modality. In most current models, human actions are represented as a set of visual features extracted from video streams or still images. The underlying action is then recognized by classification models. Meanwhile, multimodal methods combine features collected from different sources [25]. An event can be described by various informative features. For this purpose, several multimodal methods perform early or late fusion of multiple features, most simply by combining them into a larger feature vector before learning the underlying action [25].

## 3. System Design Considerations

This section describes the considerations of our robot-based education for autism therapy in clinical settings. It overviews the technology involved, applied context, and system design approach.

### 3.1. Conceptual Framework

Current rehabilitation emphasizes the importance of a theoretical framework for research development and interpretation. To advance credibility and evidence-based practice in occupational therapy, conceptual models are required to integrate evidence for systematic research and analysis of treatment outcomes [26].

The “Human Activity Assistive Technology” (HAAT) model of Cook and Hussy [27] has become a common theoretical framework for integrating assistive technology in occupational therapy. This conceptual framework guides the assessment, prescription, and result evaluation of a therapy. The functional outcome of an assistive technology system is defined as “someone (a person with a disability) doing something (an activity) somewhere (within a context)”. The HAAT model is among the earliest published assistive technology models and remains a principal framework used within the field [27].

Cook and Hussy, who proposed the original HAAT model [27], are engaged in rehabilitation engineering and occupational therapy, respectively. Both disciplines often involve assistive technology service delivery based on a conceptual model incorporating three common elements: human/person, activity/occupation, and context/environment, as proposed by Baily [28].

**Figure 1:**
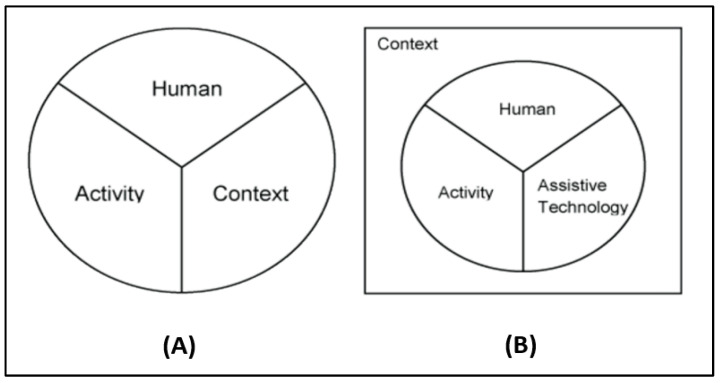
Frameworks of Bailey’s model (**A**) and the Human Activity Assistive Technology (HAAT) model (**B**).

Figure 1A,B illustrates the advancement of the HAAT model over the earlier framework of Bailey [28], achieved by incorporating two substantive changes. Bailey proposed three predictive elements of human performance: (a) human understanding, (b) activity being performed, and (c) context in which the activity is performed [29] (Figure 1A).

In the HAAT model, assistive technology is delineated as a separate and uniquely important element, having a direct and interdependent relationship with the human, activity, and contextual factors. Therefore, AT, which is not explicitly defined in Bailey’s model, is considered as a core component of the HAAT model. The HAAT model also shifts the context to a more pervasive position, as illustrated by the bounding rectangle in Figure 1B.

The context is not merely the location and physical conditions of the activity, but also involves the impacts of social, cultural, and institutional factors, and emphasizes the relevance of the activity to the individual. The four core concepts of the HAAT model—human, activity, assistive technology, and context—physical, social, cultural, and environmental factors—influence successful engagement in meaningful occupations [26].

**Figure 2:**
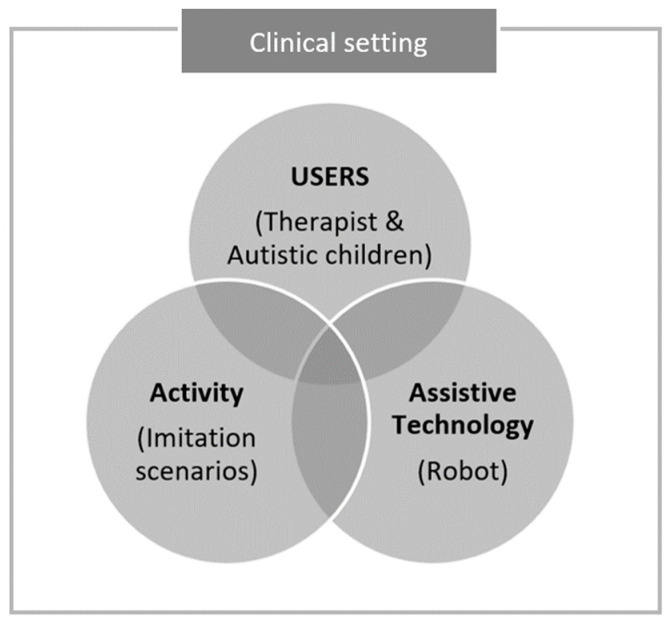
Conceptual framework of the current study.

Figure 2 presents our conceptual framework based on the HAAT model. Using robotics as the assistive technology, the framework is intended to improve the social interaction activities of children with autism.

Within the framework, we aim to illustrate variables affecting the use of robotics for children with ASD in a clinical setting, and hence improve their imitation skills. The intersection of the following four variables represents the match between user, activity, and assistive technology.

- Users: In the conceptual framework, the users are parents, family, therapists, nurses, managers, engineers, and others who will benefit from the assistive technology. In our conceptual framework, the users are therapists and children with ASD who will benefit from using robotics in a clinical setting.
- Activity: In the conceptual framework, the key purpose of using assistive technology is performing the activity that matches the individualized goal(s) of the users. In our conceptual framework, the activity is intended to improve the imitation skills of children with ASD.
- Assistive technology: It is designed to help users during a targeted activity, including various recent innovations such as virtual reality, AI, and robotics. In our conceptual framework, we employ robots for autism therapy in a clinical setting.
- Context: Assistive technology can be selected and utilized in multiple settings, such as the home, school, and community. In the present study, the context is limited to a clinical setting.

### 3.2. The Proposed Framework

**Figure 3:**
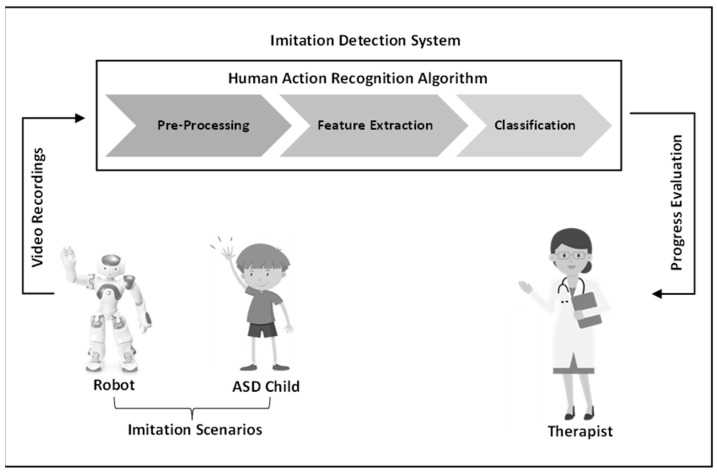
Flowchart of the proposed framework.

We propose a robot-based interactive framework for teaching imitation skills to children with autism, and analyze their interactions using a deep learning algorithm. As illustrated in Figure 3, each child attends multiple therapy sessions using a NAO robot, which involve imitation scenarios. All sessions are recorded by the robot’s camera and used in the imitation detection system.

Correct or incorrect imitation by each child is determined through video preprocessing, feature extraction, and classification of the recorded sessions. After all sessions, the therapist evaluates the learning progress and effectiveness of the robot interventions.

## 4. Materials and Methods

This section describes the materials and methods used in our study, including the NAO robot, the imitation detection system, and their integration.

### 4.1. NAO Robot

**Figure 4:**
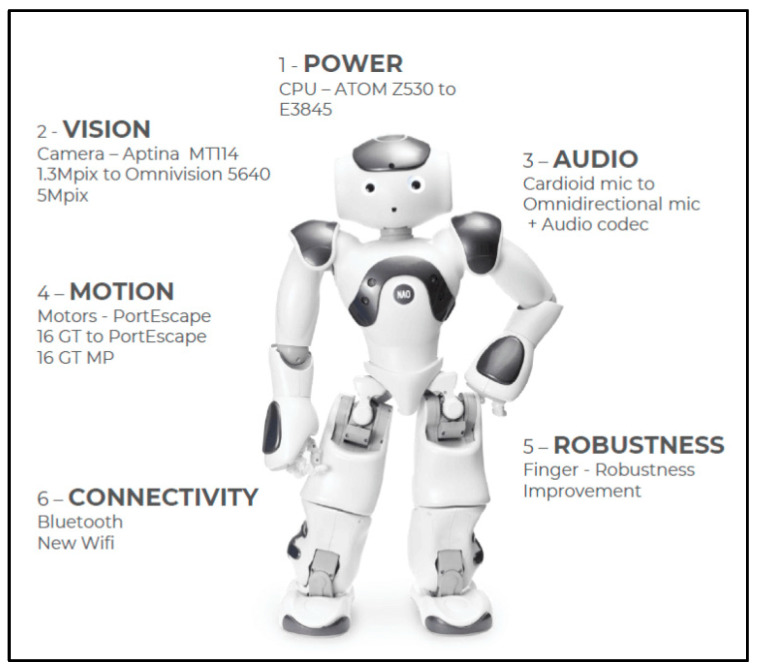
Image of the NAO 6 robot.

We deployed the Softbank Robotics NAO 6 (Figure 4), which is a humanoid robot merchandized by SoftBank Group Corporation. With 25 degrees of freedom, NAO 6 can perform most human body movements. The NAO microphones have a sensitivity of 20 mV/Pa ± 3 dB at 1 kHz and an input frequency range of 150 Hz to 12 kHz (official documentation of the Aldebaran manufacturer) [30].

This small humanoid robot can potentially provide a range of social assistance functions. Its features and capabilities, such as motricity, functionality, and affective capacities, have been studied in various contexts [31]. Furthermore, over 8000 articles returned by a Google Scholar search have demonstrated the utility of the NAO robot.

### 4.2. Imitation Detection System

The experiments and implementation of the used algorithm are detailed in our previous study [32]. In this research, we designed and developed an imitation detection system based on deep learning. Among various deep learning models, we investigated the pretrained VGG-16 model for accurate classification of human actions.

The VGG-16 architecture, developed by researchers at the University of Oxford, is a widely used convolutional neural network (CNN) for image classification, known for its simplicity and low computational complexity. It comprises 16 weight layers: 13 convolutional layers, 3 fully connected layers, and 5 max-pooling layers. Input images are resized to 224 × 224 pixels before processing. The convolutional layers utilize small 3 × 3 filters, allowing the model to learn complex features with fewer parameters, followed by ReLU activation functions. Max-pooling layers reduce spatial dimensions and mitigate overfitting by retaining the highest values in each pooling window. The final fully connected layer employs a Softmax activation function to generate class probabilities.

In our work, we utilized the pretrained VGG-16 model due to its established performance in image classification. The proposed models were evaluated on the KTH benchmark dataset, which contained six types of human actions: walking, jogging, running, boxing, hand waving, and hand clapping.

For data preprocessing, videos were categorized into “clapping” and “non-clapping” folders, and frames were extracted using OpenCV, resized to 224 × 224 pixels. Data augmentation techniques, such as translation and horizontal flipping, were applied to the training set to combat overfitting.

Feature extraction involved loading the VGG-16 model and adding dropout layers to prevent overfitting. A flatten layer converted the multidimensional input to one dimension, followed by a dense layer with a sigmoid activation function for binary classification. The VGG-16 model’s parameters were frozen to maintain the pretrained weights.

We also compared the performances of our models with those of classical classifiers (single-frame convolutional neural network (CNN), K-Nearest Neighbor, Support Vector Machine, and Random Forest). The performance metrics are summarized in Table 1, which includes key measures such as accuracy, precision, recall, and F1-score.

The results indicate that RF and VGG-16 models emerge as the top performers, with RF achieving the highest accuracy (98%) and a remarkable recall rate (99%), indicating its effectiveness in correctly identifying positive instances. VGG-16 matches RF in accuracy while achieving a perfect recall of 100%, demonstrating its capability to capture all relevant cases. In contrast, the Single-frame CNN, while exhibiting high precision (97%), shows a lower recall (85%) and accuracy (91%), suggesting it may overlook some positive instances. Overall, the results indicate that RF and VGG-16 are the most reliable choices for applications requiring high sensitivity and precision, while the SVM and KNN also deliver strong performance, albeit with some trade-offs in recall and overall effectiveness.

The VGG-16 model, while effective, is sensitive to behavioral variations that can impact its performance and assessment validity. Differences in posture, facial expressions, or contextual actions may lead to misclassifications or reduced accuracy, especially if the training data lack diversity. This can result in skewed evaluations, either overestimating or underestimating a child’s abilities [32]. Although we utilized the KTH dataset, which includes hand-clapping actions performed multiple times by 25 subjects across four different scenarios to ensure variation, it is still crucial to address these behavioral differences. Considering them during both training and evaluation will enhance the model’s robustness and ensure valid assessments.

In our experiment, the VGG-16 model was employed as the deep learning model for assessing successful or unsuccessful imitations of the robot by the children with ASD. Our system inputs recorded videos and preprocesses them to generate individual frames. The features in the frames are then extracted and classified. Owing to its rapid and stable classification capabilities, the CNN-based VGG-16 has become a preferred algorithm for gesture detection in image classification tasks. Importantly, the network can learn features without requiring manual feature extraction, and can be trained on raw images to automatically create feature extraction classifiers [33].

### 4.3. Robot Implementation

To implement our proposed scenario based on the NAO robot, we first set up the robot by pressing its chest button to access the NAO page. We then connected the robot to a laptop by entering the IP address. Thereafter, we configured the Wi-Fi connection settings, account settings, language, and time zone of the robot.

The robot was programmed using a multiplatform desktop application called Choregraphe, which allows for the creation of complex behaviors (e.g., robot–human interactions and dancing) through drag-and-drop features and Python scripting [34]. To create an action, we selected boxes from the Box Libraries panel, which contained all of the necessary elementary boxes for initial behaviors such as movement, speech, and sensing. The boxes were then connected to define the desired sequence of actions. For instance, an action might begin with a “Play Sound” box to make the robot talk or to upload an .OGG audio file, followed by a sequence of movement boxes to define clapping motions and an ending pose.

**Figure 5:**
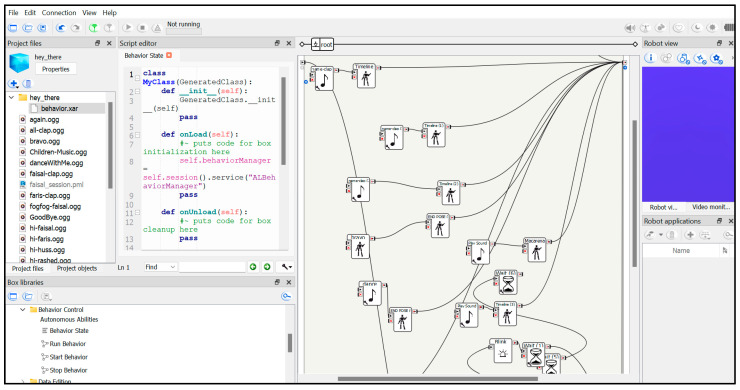
Robot implementation in Choregraphe 2.8.7.

Each box provides options for modifying various parameters, either through a Python code or through a dialogue window; e.g., modifying the sound volume or movement speed. Finally, the program was started by pressing the play button or using the voice command feature, through which the robot listens and begins the program. Figure 5 shows a program within the Choregraphe interface.

## 5. Experimental Design

For testing and validating the proposed approach, the proposed framework must be evaluated in a clinical and real-world context/setting. This section presents the experimental design (experimental procedures and protocols of the imitation scenarios involving the children with autism and robot), along with details on participants, data collection, and data analysis.

The goal of our proposed system was to assist therapists using robotics during therapy sessions involving children with autism, mainly to improve the imitation skills of the children. To assess the effectiveness of the therapy, we analyzed and evaluated the progress of the children with autism during imitation training in each session.

### 5.1. Collaboration Process

**Figure 6:**
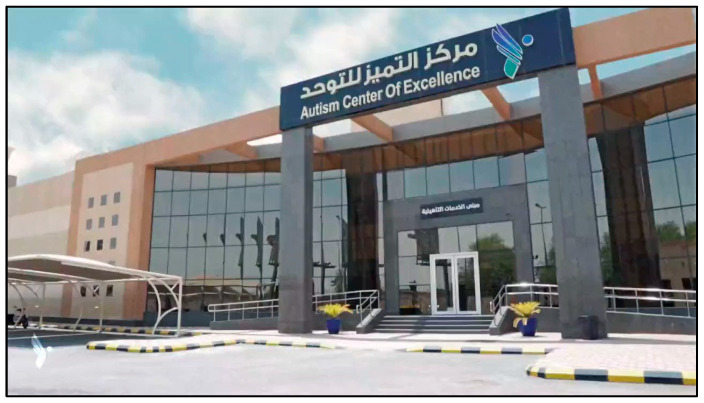
Autism Center of Excellence (ACE).

We collaborated with the Autism Center of Excellence (ACE) (Figure 6), which provided essential resources and therapy specialists for our experiment. The ACE was established in Riyadh as part of a corporate social responsibility initiative involving the Ministry of Human Resources and Social Development and Saudi Central Bank [35].

During our initial meeting, we introduced the project and discussed the potential benefits of using robotics as an educational tool with the chief operating officer and a team of therapists at the ACE. We explored the center’s teaching protocols, emphasizing imitation as the primary skill for children. Subsequent meetings included a tour of the facilities, where we observed various activities and therapy sessions, gaining insights into the methods used by the therapists and challenges faced by children.

We also discussed strategies for training and monitoring progress in imitation skills, and developed a protocol by which the NAO robot can replicate therapeutic scenarios. Therapists at the ACE identified hand clapping as the primary imitation skill to focus on, noting that this task presents inherent challenges for children with ASD and serves as an effective starting point for robot-assisted intervention therapy. By targeting this specific action, the protocol aims to establish a measurable and achievable goal for the children, thereby facilitating their engagement and learning during therapy sessions.

Finally, during a remote meeting, we finalized the experimental protocol, established the start date, selected the sample of children, and determined the number of sessions to be conducted, ensuring alignment in the execution of the experiment.

### 5.2. Ethical Statement

The experimental protocol was reviewed and approved by the Institutional Review Board of the College of Medicine at King Saud University (reference number 22/0154/IRB; research project number E-21-6377). The participants’ parents were informed of this study’s objectives, implications of their involvement, and potential benefits and risks.

Prior to enrolment in this study, each participant or their legal guardians were required to provide consent of their voluntary and free-willed participation. The participants were also informed that they could withdraw from this study at any moment without any consequence. In addition, they were informed that this study’s findings would be released later and their confidentiality would be protected at all times during this study.

### 5.3. Participants

To partake in this experiment, volunteers were required to meet specific criteria: (a) a diagnosis of ASD, (b) age of 4 years or older, and (c) ability to perform simple imitation movements. This criterion was crucial for assessing the impact of the robot-assisted intervention on the speed and efficiency of their learning during therapy sessions, and (d) no diagnosis of attention deficit/hyperactivity disorder (ADHD) or low levels of ADHD symptoms. The last criterion was necessary because the robot is immobile and cannot record and track children beyond a limited range.

In Ref. [8], a systematic literature review revealed that the number of participants in studies using robots for therapy with children with ASD varied from 2 to 45. Additionally, the number of sessions ranged from 1 to over 30, depending on the study design.

In this study, seven children with a DSM-5 [1] diagnosis of autism at the Autism Center of Excellence—with ages from 5 to 9 years old (M = 7); one female and six males—were involved in the study. All children were verbal (able to speak a few words) and met the ADOS-2 [36] criteria for autism (Table 1).

### 5.4. Protocol

One of the key diagnostic indicators of ASD is difficulty with early imitation skills. Lack of imitation is a distinct characteristic of the disorder. However, children with ASD with increased imitation abilities can better develop their skills in various areas [37].

To improve the imitation skills of children with ASD, we developed a protocol comprising 30 sessions spread over three weeks, with a frequency of two to three days per week. Each child was expected to attend between three to six sessions, accounting for absences or time conflicts with their core sessions and our sessions.

Two types of sessions were conducted: initial sessions and actual sessions. During the initial sessions (each lasting for three to five minutes), the children were introduced to the robot and engaged in simple interactive scenarios such as welcoming, dancing, and farewell. These sessions enabled the participants to familiarize themselves with the robot and the researchers to exclude participants exhibiting fear or aggressive behavior, who would be unsuitable for the experiment.

The initial sessions were followed by the actual sessions, which were thoroughly customized based on information collected from the therapist responsible for each child. As the sessions were tailored to the specific preferences of the child, they were expected to be enjoyable and effective. During each actual session, the children engaged in several uniquely created scenarios with suitable activities such as asking questions, dancing, and playing their preferred music. To personalize the experience, the children were directly addressed by their names when introduced to each imitation task; for instance, an artificially generated female voice [38] might instruct “Muhammad, clap your hands” in the Arabic language (see Table 2).

**Figure 7:**
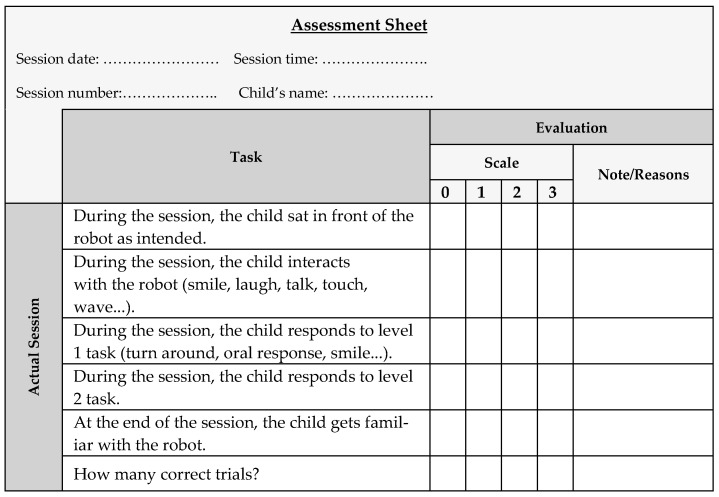
Assessment sheet used in the experiments.

Each child was given ten clapping attempts, and the success of the session was evaluated by the therapist on an assessment sheet previously designed by the same therapist. Each item on the assessment sheet (Figure 7) was rated on a scale ranging from 0 (strong disagreement) to 3 (strong agreement).

### 5.5. Experimental Setting

Experiments were performed in a small, well-lit room measuring 3 × 4 m within the ACE. The number of items in the room was minimized to suppress distractions.

**Figure 8:**
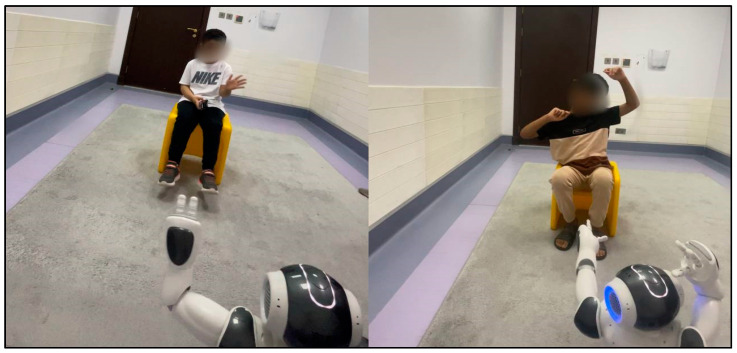
Images taken during the initial sessions of the experiment.

As shown in Figure 8, the NAO robot was placed directly in front of the child, who was seated either on a chair or on the ground. The child-to-robot distance was carefully selected to protect the child from injury if the robot fell.

The sessions were conjointly controlled by the researcher and the child’s therapist. The robotic applications were launched through an interface on the laptop connected to the robot via Wi-Fi. The sessions were accurately recorded by two cameras: a camera built into the robot and a backup mobile phone camera with a lighting stand placed behind the robot. The images in Figure 8 were taken during the initial sessions, when the robot greeted the children and played music.

### 5.6. Data Collection

The experimental sessions were carefully recorded for analyzing clapping imitations. The recordings captured by the two cameras were subsequently subjected to preprocessing, feature extraction, and action/motion classification.

**Figure 9:**
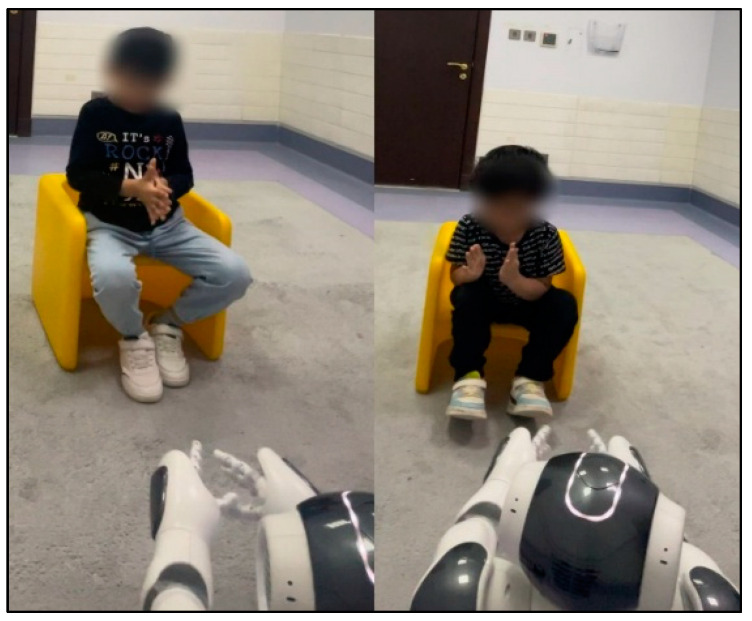
Recorded frames.

The primary aim of this analysis was to evaluate the training of imitation skills in children with autism. Therefore, the entire body of the child was accurately captured in the recordings to facilitate imitation detection by the algorithm. Two suitably detected frames are shown in Figure 9. Overall, the high-quality video recordings enabled the accurate analysis of clapping imitation.

## 6. Results and Discussion

This section presents the experimental outcomes, including the imitation results of each child acquired from the proposed imitation detection system using the VGG-16 model. Moreover, the learning progress of each child was evaluated using the Verbal Behavior Milestones Assessment and Placement Program (VB-MAPP) assessment tool [39]. The overall experience of the sessions was determined from manual assessments by the therapists at the ACE.

By adopting the VB-MAPP assessment tool, we evaluated whether each child had imitated the robot’s actions, acquired the skill, and applied it to various contexts (i.e., generalized the skill). The VB-MAPP assessment tool, which is designed to evaluate and track the development of language and social skills of children with autism, serves as a curriculum guide, criterion-referenced assessment tool, and skill-tracking system [39]. Imitation skills were considered to be mastered if the child achieved 80% to 100% correct trials across three independent sessions, as done in a previous study [40].

Table 3 presents the number of sessions undertaken by each child and the corresponding percentage of correct trials detected by the imitation detection system. A comparison between the system’s outcomes and actual results confirmed the high accuracy of the system. The obtained data revealed that most of the children effectively acquired the clapping imitation skill, although two children showed no substantial improvement. Notably, C1, C3, and C6 achieved the highest percentage of correct trials throughout their sessions, indicating that these children mastered the task well. Moreover, the acquisition of the imitation skill improved over time, as demonstrated by C2’s progress.

To assess whether robots can effectively improve the imitation skills of children with ASD, we compared the results of two groups of children diagnosed with autism. One group had received training for imitation skills from a robot; the other group had received standard training from therapists. Although only two of the seven children participating in our robot-assisted training failed to acquire imitation skills, three of the seven children in the non-robotic-therapeutic group struggled to master the same skills (Table 4). To ensure a balanced distribution of sessions between the two groups and the same number of sessions for all children (the number of sessions varied among the children), we selected only the first three sessions for the comparative analysis (Table 5).

As shown in Table 5, the results demonstrated a marked difference between the two training approaches. The robot-assisted group exhibited an average improvement of 17.14%, with several children achieving substantial gains in their imitation skills. For instance, while two children did not master the skills, the majority showed significant progress, with one child achieving a maximum level of 100%. In contrast, the non-robotic therapeutic group recorded a modest average improvement of 2.86%, indicating minimal gains in imitation skills. Most children in this group struggled to enhance their performance, with only a few showing slight improvements. These findings suggest that robot-assisted interventions may provide a more effective and engaging method for developing imitation skills in children with ASD compared to traditional therapeutic approaches.

The completion of each session was followed by feedback and comments from specialists. We also collected response data from the assessment sheet as outlined in Section 5.4. We emphasize that our assessment was general and considered only the children’s overall interaction with the robot, which was not specific to clapping skills. To track progress, we calculated the progress scores from the assessments of the specialists using a method described in Ref. [41].

**Figure 10:**
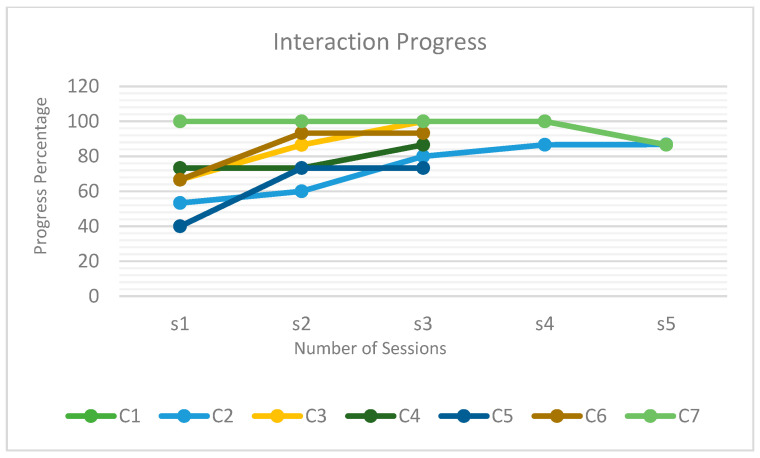
Trends of assessment scores for the sessions.

As shown in Figure 10, the children progressively interacted with and accepted the robot as the sessions proceeded. However, as mentioned previously, the psychological and physical well-being of the children were also important. For instance, the interaction of C7 increased during each session, but decreased during the final session, when progress was impeded by illness.

Valuable feedback received from the specialists, such as “the children appear excited and eager to see the robot after each session”, “they follow the robot’s commands attentively”, and “we have not previously witnessed such enjoyment during sessions”, collectively confirmed the effectiveness of the robot. In this context, we conclude that robots can substantially enhance the learning experience under the supervision of therapists.

The experiment results indicate a potential therapeutic role of intelligent social robots in enhancing the imitation skills of children with ASD.

## 7. Conclusions

This study examined the potential of robotics to improve imitation skills in children with autism and to support therapists during therapeutic sessions. We developed a deep learning-based imitation detection system for human activity recognition, focusing on detecting clapping imitation. Utilizing the NAO robot, we created scenarios to train imitation skills in children with autism, capturing their interactions through video analysis.

Our findings suggest that intelligent social robots can be valuable tools in robot-assisted therapy, aiding children with ASD in achieving their therapeutic and educational goals. By enhancing imitation skills, these robots can significantly contribute to the social, emotional, and cognitive development of children with ASD, underscoring the potential for interdisciplinary research and development in this area.

However, our study has limitations, including a relatively small participant pool and a limited number of sessions, which restricts the ability to draw broader conclusions about the effectiveness of robot-assisted therapy.

For future work, we recommend two key actions: first, expanding the participant pool to include a larger and more diverse group, comprising both robot-assisted and traditional therapy groups, to strengthen the evaluation of the framework’s effectiveness; second, conducting more sessions across participants to measure progress comprehensively. Longer-term session analyses are essential for understanding the robot’s effects on learning over time. Additionally, objective metrics such as gaze tracking, facial expressions, or motion detection sensors could be employed to measure children’s engagement. Future research could also explore the generalization of imitation skills beyond the robotic context and develop more robust algorithms to detect motion and assess imitation sensitivity to behavioral variations.

We conclude with three potential directions for future research: (i) increasing the number of clinical sessions and expanding the sample size to assess the effectiveness of our proposed framework, (ii) enhancing the robot’s capabilities and the imitation detection system for real-time analysis and online accessibility, and (iii) improving the imitation detection system to recognize a broader range of movements while increasing the robot’s level of automation to reduce the need for human control.

## Figures and Tables

**Table 1:** Performance metrics.

Algorithm	Accuracy (%)	Precision (%)	Recall (%)	F1 (%)
SVM	93	95	92	93
RF	98	98	99	98
KNN	97	99	96	97
Single-frame CNN	91	97	85	90
VGG-16	98	96	100	98

**Table 2:** Details of the study participants.

Child	Age	Gender	Autism Level	ADHD
C1	5	Male	2	No
C2	6	Male	2	Yes
C3	6	Male	2	No
C4	7	Male	2	Yes
C5	8	Male	3	No
C6	8	Male	3	No
C7	9	Female	3	No

**Table 3:** Experimental protocol.

Objective	To improve imitation skills in children with ASD
Experiment Date and Period	2nd of May 2023–29th of May 2023 (four weeks)
Location	Autism Center of Excellence
Target Participant	Children with ASD
Number of Sessions	3 to 6 sessions/child
Session Duration	8–10 min
Child Age Group	5–9
Number of Children	7
Experiment Protocol	The robot performs tasks in front of the child. The tasks are divided into two levels: L1: The robot will call the child’s name. L2: The robot will do hand clapping. The child completing the tasks will be videotaped during the whole session. In addition, the therapist and the researcher will evaluate the child interaction/imitation on an assessment sheet. After conducting all sessions, the child’s progress will be evaluated in terms of number of correct trials.
Experiment Tasks (Initial and Actual)	Initial tasks: free playing and dancing. Actual tasks: hand clapping imitation.

**Table 4:** Analysis of the experimental results.

Child	Percentage of Correct Trials					Mastered Skill
	S1	S2	S3	S4	S5	
C1	90%	100%	100%	-	-	Y
C2	20%	60%	70%	80%	80%	N
C3	80%	100%	90%	100%	-	Y
C4	80%	80%	90%	-	-	Y
C5	10%	30%	20%	-	-	N
C6	90%	80%	100%	-	-	Y
C7	90%	80%	80%	50%	-	Y

**Table 5:** Comparison of imitation skill mastery by children on robot and non-robotic-therapeutic sessions.

Robot/Non-Robotic-Therapeutic Sessions	Child	Percentage of Correct Trials			Mastered Skill
		S1	S2	S3	
Robot Sessions	C1	90%	100%	100%	Y
	C2	20%	60%	70%	N
	C3	80%	100%	90%	Y
	C4	80%	80%	90%	Y
	C5	10%	30%	20%	N
	C6	90%	80%	100%	Y
	C7	90%	80%	80%	Y
Non-Robotic- Therapeutic Sessions	C8	100%	100%	60%	N
	C9	100%	100%	100%	Y
	C10	90%	30%	90%	N
	C11	90%	100%	100%	Y
	C12	100%	100%	100%	Y
	C13	90%	80%	80%	Y
	C14	0%	10%	10%	N

## Data Availability

KTH dataset used in this study is available: https://www.csc.kth.se/cvap/actions/ (accessed on 19 December 2024).
